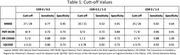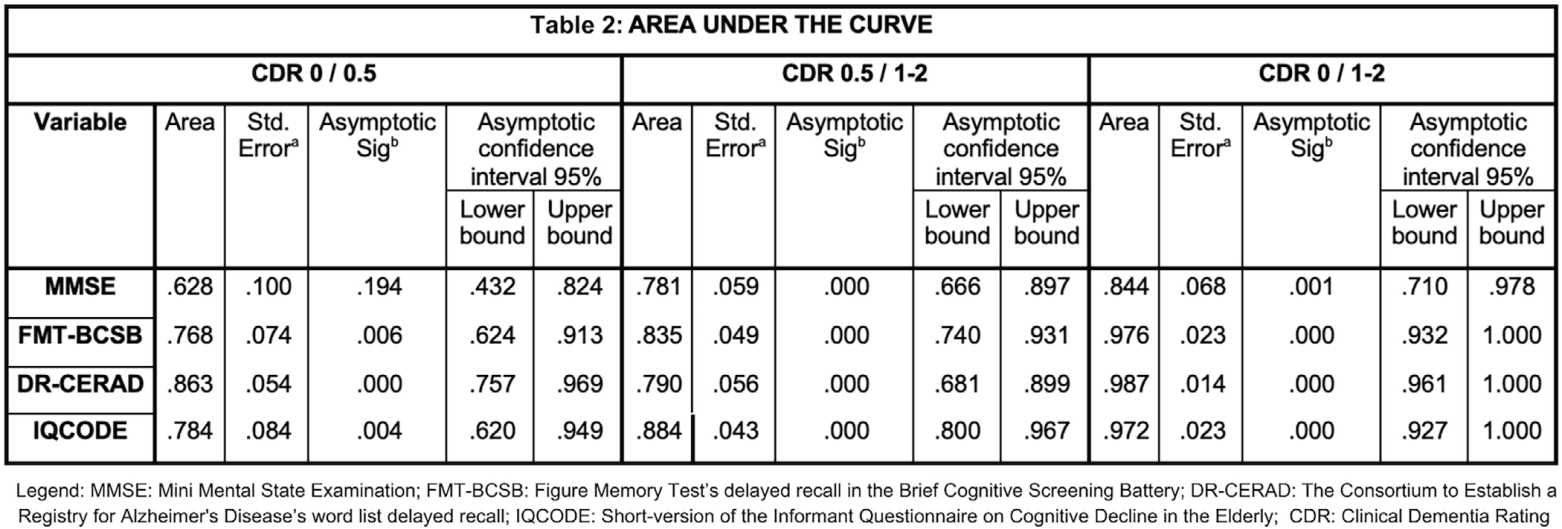# Accuracy of cognitive and functional screening tests to Clinical Dementia Rating in an Outpatient Memory Clinic

**DOI:** 10.1002/alz.092908

**Published:** 2025-01-03

**Authors:** Giovanna Correia Pereira Moro, Aline Siqueira de Souza, João Vitor da Silva Viana, Gabriela Tomé Oliveira Engelmann, Ivonne Carolina Bolaños Burgos, Bruna Fugêncio Dias, Carolina Mizerani Couto Moreira, Karoline Freire Kosac, Erika de Oliveira Hansen, Marco Aurélio Romano‐Silva, Bernardo de Mattos Viana, Maria Aparecida Camargos Bicalho

**Affiliations:** ^1^ Undergraduate Medicine, Federal University of Minas Gerais, Belo Horizonte, Minas Gerais Brazil; ^2^ Sciences Applied to Adult Health Postgraduate Program, School of Medicine, Universidade Federal de Minas Gerais (UFMG), Belo Horizonte, Minas Gerais Brazil; ^3^ Cog‐Aging Research Group, Universidade Federal de Minas Gerais (UFMG), Belo Horizonte, Minas Gerais Brazil; ^4^ Undergraduate medicine, Faculty of Medicine, Universidade Federal de Minas Gerais (UFMG), Belo Horizonte, Minas Gerais Brazil; ^5^ Molecular Medicine Postgraduate Program, School of Medicine, Universidade Federal de Minas Gerais (UFMG), Belo Horizonte, Minas Gerais Brazil; ^6^ Neurotec R National Institute of Science and Technology (INCT‐Neurotec R), Faculty of Medicine, Universidade Federal de Minas Gerais (UFMG), Belo Horizonte, Minas Gerais Brazil; ^7^ Older Adult’s Psychiatry and Psychology Extension Program (PROEPSI), School of Medicine, Universidade Federal de Minas Gerais (UFMG), Belo Horizonte, Minas Gerais Brazil; ^8^ Cog‐Aging Research Group, Belo Horizonte, Minas Gerais Brazil; ^9^ Molecular Medicine Postgraduate Program, Faculty of Medicine, Universidade Federal de Minas Gerais (UFMG, Belo Horizonte, Minas Gerais Brazil; ^10^ Department of Psychiatry, School of Medicine, Universidade Federal de Minas Gerais (UFMG), Belo Horizonte, Minas Gerais Brazil; ^11^ Department of Internal Medicine, School of Medicine, Federal University of Minas gerais, Belo Horizonte, Minas Gerais Brazil; ^12^ Geriatrics and Gerontology Center Clinical Hospital of University of Minas Gerais, Belo Horizonte, Minas Gerais Brazil

## Abstract

**Background:**

Short screening tools designed to detect cognitive impairment are important for clinical and research. The Clinical Dementia Rating (CDR) is the main used categorization system, which classifies from no dementia, to questionable dementia/mild cognitive impairment (MCI) and three severities of dementia.

**Objective:**

To conduct an accuracy analysis of different short screening tests to predict CDR scores on a cohort of a Memory Clinic.

**Methods:**

This is a cross‐sectional study, using data from the Cog‐Aging cohort study in 2023. Participants go through a comprehensive clinical, neuropsychological and neuroimaging assessment to determine the diagnosis and CDR. Eighty participants were recruited: 11 controls, 43 MCI, and 26 Dementia. Mini Mental State Examination (MMSE), Figure Memory Test’s delayed recall in the Brief Cognitive Screening Battery (FMT‐BCSB), The Consortium to Establish a Registry for Alzheimer’s Disease’s word list delayed recall (DR‐CERAD) and Short‐version of the Informant Questionnaire on Cognitive Decline in the Elderly (IQCODE) were used to distinguish the CDR scores. Participants were classified based on CDR (0; 0.5; and 1‐2). We performed a Receiver Operating Characteristic (ROC) curve and cut‐off values defined by Youden’s J statistic comparing: CDR 0 x CDR 0,5; CDR 0,5 x CDR 1‐2; CDR 0 x CDR 1‐2. This study was approved by the ethics committee of UFMG.

**Results:**

Participants had a mean age of 77.7 yr. (SD 6.9) and median 6.1 years of education (IQR 5.25). The DR‐CERAD had the best area under the curve (AUC) (0.863) to distinguish CDR 0 to 0.5, with 88% sensitivity with cut‐off points of 5 / 6. Comparing CDR 0.5 to 1‐2, IQCODE had the largest AUC (0,884) with 92% sensitivity with cut‐off ≥ 3,78. Comparing CDR 0 to 1‐2, the DR‐CERAD had the largest AUC (0.987), with 88% sensitivity with cut‐off points of 3 / 4.

**Conclusion:**

These findings suggest that DR‐CERAD is the most accurate to distinguish MCI to normal cognition, and normal cognition to dementia in this sample. IQCODE presented as the best to distinguish MCI to dementia. These are preliminary results and more studies with years of education and a larger sample are necessary.